# A Rare and Severe Multisystem Cascade of AKI, ARDS, and Septic Shock Leading to Acalculous Cholecystitis in a Young Scrub Typhus Patient: A Case Report From Nepal

**DOI:** 10.1002/ccr3.71632

**Published:** 2025-12-04

**Authors:** Prabhat Kaphle, Maya Upadhyaya, Sarjan Shrestha, Amrita Shrestha, Dhanlaxmi Giri, Kamal Hamal, Liladhar Ojha

**Affiliations:** ^1^ National Health Action Force Nepal Dhading Nepal; ^2^ Surkhet Provincial Hospital Surkhet Nepal; ^3^ Bhojpur District Hospital Bhojpur Nepal

**Keywords:** acalculous cholecystitis, acute kidney injury, scrub typhus, septic shock

## Abstract

Scrub typhus, caused by *Orientia tsutsugamushi* , is an acute febrile illness prevalent in Nepal with a wide spectrum of clinical presentations. Severe forms can lead to multiorgan dysfunction, including acute kidney injury (AKI), acute respiratory distress syndrome (ARDS), septic shock, and rarely, acalculous cholecystitis. We report a case of a previously healthy 30‐year‐old female from Nepal presenting with fever, jaundice, and epigastric pain. Clinical evaluation revealed hemodynamic instability, respiratory distress, and an eschar on the lower extremity. Laboratory and imaging workup showed AKI, elevated liver enzymes, positive IgM and IgG for *O. tsutsugamushi* , gallbladder wall thickening without stones, and chest radiograph findings consistent with ARDS. She was diagnosed with scrub typhus complicated by AKI, ARDS, septic shock, and acute acalculous cholecystitis. The patient was treated promptly with doxycycline and supportive intensive care, including oxygen therapy, intravenous fluids, and antibiotics. Clinical and laboratory improvement ensued, and she was discharged in stable condition after 2 weeks of hospitalization. This case highlights the potential for severe multisystem complications of scrub typhus, including the rare occurrence of acalculous cholecystitis secondary to systemic vasculitis and shock. Early recognition and timely management in endemic regions are critical to improving outcomes.

## Key Clinical Message

This case underscores the importance of considering scrub typhus as a cause of unexplained sepsis and abdominal pain in endemic areas, since early antimicrobial therapy and supportive care can reverse otherwise fatal multisystem failure.

**Abbreviations**
ABG: arterial blood gas
AKI: acute kidney injury
ALT: alanine aminotransferase
ARDS: acute respiratory distress syndrome
AST: aspartate aminotransferase
CBC: complete blood count
DIC: disseminated intravascular coagulation
ELISA: enzyme‐linked immunosorbent assay
GB: gallbladder
ICU: intensive care unit
IgG: immunoglobulin G
IgM: immunoglobulin M
NIV: non‐invasive ventilation
PT/INR: prothrombin time/international normalized ratio
RA: room air
RR: respiratory rate
SPO_2_
: oxygen saturation
TB: total bilirubin
WBC: white blood cell count

## 1 Introduction

Scrub typhus is an acute febrile illness caused by *Orientia tsutsugamushi* , commonly found in Nepal. Presentation varies from subclinical to multiple organ failure to death, with acute or chronic onset of fever with chills, malaise, headache, myalgia, rash, eschar, and lymphadenopathy. Complications include acute kidney injury (AKI), acute respiratory distress syndrome (ARDS), meningoencephalitis, myocarditis, jaundice, pneumonitis, septic shock, pericarditis, and disseminated intravascular coagulation (DIC) [1, 2].

Sepsis is defined as life‐threatening acute organ dysfunction secondary to infection [3]. One of the most crucial consequences of sepsis includes acalculous cholecystitis. It is an inflammation of the gallbladder that occurs in the absence of gallstones. It accounts for 5%–15% of cases of cholecystitis, although calculous cholecystitis is the most common cause. Acalculous cholecystitis results from stasis of the gallbladder, cholestasis, and hypoperfusion due to various causes like prolonged fasting, total parenteral nutrition, trauma, critical illness, or rapid weight loss [4, 5]. *Orientia tsutsugamushi* has been associated with acute acalculous cholecystitis in otherwise healthy adults in various literature. Infection with scrub typhus can cause hypoperfusion leading to ischemia, necrosis, or rupture of the gallbladder, eventually leading to peritonitis if delayed and managed inappropriately [6].

Here, we report a case of a young healthy adult from Nepal without any associated comorbidities and past history of similar episodes. She presented to this center with multi‐system involvement and septic shock contributing to rare acute acalculous cholecystitis. The patient was timely referred to the tertiary center, clinically evaluated, treated, and recovered early, and is recorded in compliance with CARE Guidelines [7].

## 2 Case Report

### 2.1 Patient Information

A 30‐year‐old previously healthy, non‐gravid, non‐alcoholic female, farmer by occupation, presented to the Surkhet Provincial Hospital ER department with complaints of fever and yellowish discoloration of the body for 5 days. Before hospital presentation, she experienced epigastric pain, nausea, anorexia, and loose stools, and was managed in a clinic conservatively. She denied abdominal pain radiating to the back, steatorrhea, or malnutrition. The patient had no underlying comorbidities and was not on any regular medications.

### 2.2 Clinical Findings

She was referred to the hospital ER after clinical deterioration. Initially in the ER, she was conscious and mentally alert, but her vitals were unstable. Her BP was 90/60 mmHg, PR was 166 bpm, temperature was 100°F, RR was 46 breaths/min, and SPO_2_ was 85% in RA.

**FIGURE 1:**
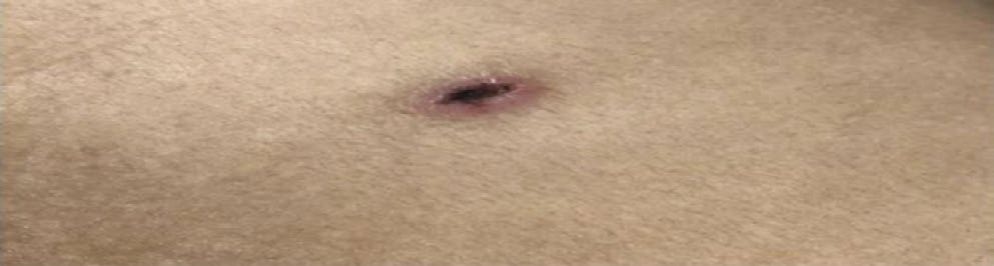
Eschar located on the medial aspect of the left lower extremity, a characteristic skin lesion in scrub typhus.

On general examination, she was ill‐looking with an eschar present on the medial aspect of the left lower extremity (Figure 1). Respiratory examination revealed bilateral decreased breath sounds and basal crepitus. Abdominal examination showed non‐radiating right epigastric pain with a positive Murphy's sign but no guarding, rigidity, palpable mass, or organomegaly. Bowel sounds were intact.

### 2.3 Diagnostic Assessment

Routine blood investigations, random blood glucose tests (72 mg/dL), PT/INR (16 s/1.23), serology tests, serum lipase (83 units/L), and echocardiograms were within normal limits, but arterial blood gas analysis indicated type 1 respiratory failure. CBC showed neutrophilia with a normal total blood count. There was a low platelet count, hypoalbuminemia, high bilirubin and liver enzymes, and increased creatinine levels (Table 1). A serological test showed positive IgG and IgM *Orientia tsutsugamushi* with negative malaria, Widal, and dengue rapid tests.

**TABLE 1:** Laboratory investigations on Days 1, 2, and 3.

Laboratory parameter	Day 1	Day 2	Day 3	Normal range
Leukocytes (/mm^3^)	7600	—	—	4000–11,000
Total bilirubin (mg/dL)	13.77 ↑	15.7 ↑	16.2 ↑	0.2–1.2
Direct bilirubin (mg/dL)	8.28 ↑	8.17 ↑	7.8 ↑	0–0.4
Total protein (g/dL)	6.05	4.87 ↓	5.1 ↓	6.0–8.0
Albumin (g/dL)	2.43 ↓	2.38 ↓	2.16 ↓	3.5–5.5
Alkaline phosphatase (U/L)	552 ↑	595 ↑	1132 ↑	42–98
AST (IU/L)	223 ↑	186 ↑	169 ↑	10–40
ALT (IU/L)	91 ↑	88 ↑	73 ↑	10–35
Creatinine (mg/dL)	2.15 ↑	1.69 ↑	0.87	0.4–1.4
Urea (mg/dL)	163 ↑	139 ↑	50 ↑	15–45
*Orientia tsutsugamushi* IgM/IgG	Positive	—	—	Negative

*Note:* ↑ indicates above normal range; ↓ indicates below normal range.

### 2.4 Radiological Findings

**FIGURE 2:**
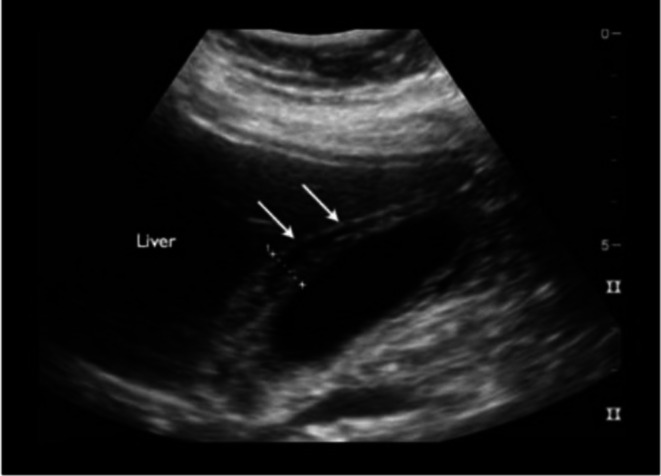
Longitudinal ultrasound view of the gallbladder demonstrating circumferential thickening of the anterior wall with mild pericholecystic fluid collection, indicative of acute acalculus cholecystitis (white arrows).

Ultrasound imaging showed edematous circumferential thickening of the gallbladder wall with minimal pericholecystic fluid collection without gallstones, with normal pancreatic shape and size, suggestive of acute acalculous cholecystitis (Figure 2).

**FIGURE 3:**
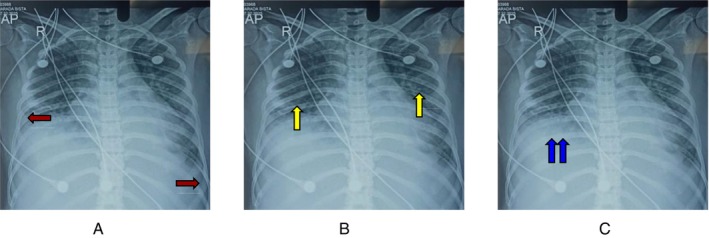
(A) Chest X‐ray demonstrating bilateral pleural effusions (red arrows). (B) Chest X‐ray demonstrating bilateral pulmonary infiltrates (yellow arrows). (C) Chest X‐ray demonstrating area of atelectasis (blue arrows).

A chest X‐ray revealed bilateral pleural effusion (Figure 3A), atelectasis (Figure 3C), and bilateral pulmonary infiltration (Figure 3B).

### 2.5 Treatment History

She was admitted to the ICU and treated with parenteral fluids, parenteral antipyretics, and parenteral antibiotics. Medications included doxycycline 5 mg/kg twice a day for 7 days and piperacillin and tazobactam 4 g thrice a day for 5 days, then changed to ceftriaxone 1 g twice a day for 5 days. She was monitored on intermittent non‐invasive ventilation (NIV) for the first 4 days and then switched to O_2_ delivery via facemask at the rate of 4–6 L/min (Table 2). Nebulization with bronchodilators and chest physiotherapy was performed simultaneously. Her condition improved, and she was discharged after 2 weeks of ICU stay (Table 2). The patient was stable, and lab findings were normal at the time of discharge.

**TABLE 2:** Case timeline throughout hospital stay.

Day	Event/activity
Day 1	Admitted to ER. Unstable vitals. Eschar noted. Labs confirmed AKI, liver dysfunction, and positive IgM/IgG. Treatment with IV doxycycline, fluids, and NIV was initiated.
Day 2	USG confirmed acalculous cholecystitis. CXR showed an ARDS pattern.
Days 5	Antibiotics de‐escalated to ceftriaxone. Switched to mask O2.
Day 14	Discharged, labs normalized.
Day 21 (follow‐up)	Stable, no complications.

### 2.6 Follow‐Up and Outcomes

After one week of follow‐up, the patient had no significant issues. She is doing well.

## 3 Discussion

Scrub typhus is well established as a severe rickettsial disease that is caused by the obligate, intracellular bacterium *O. tsutsugamushi* , which is maintained in nature by trans‐ovarian transmission in trombiculid mites. Human involvement occurs accidentally when they get bitten by infected trombiculid mite larvae (chiggers), leading to inoculation of organisms into the skin. The clinical spectrum and mechanisms underlying complications—especially acute acalculous cholecystitis, acute kidney injury (AKI), acute respiratory distress syndrome (ARDS), obstructive jaundice, and shock—are multifactorial, with endothelial damage and systemic inflammatory responses at the core.

### 3.1 Pathophysiology of Complications

#### 3.1.1 Acalculous Cholecystitis

Acalculous cholecystitis, while rare, is increasingly reported in patients with severe scrub typhus. Its underlying mechanism is believed to be systemic vasculitis or perivasculitis, caused by *O. tsutsugamushi* acting on the small vessels, including those supplying the gallbladder [1]. Orienta Tsutsugamushi shows tropism to several host cells, including endothelial cells, causing endothelitis. The dissemination occurs within macrophages, ultimately reaching the human gallbladder [6]. The vasculitis leads to ischemia, bile stasis, disruption of microcirculation, and ultimately, inflammation and necrosis of the gallbladder wall even in the absence of gallstones [4]. Severe illness and systemic shock exacerbate this process by further compromising gallbladder perfusion. Literature suggests old age, elevated creatinine, low albumin, and leukocytosis as risk factors for the development of various complications in scrub typhus [8]. Pathological findings typically show a thickened gallbladder wall and microvascular changes, sometimes without overt neutrophilic infiltration, highlighting the vascular rather than purely infectious nature of injury [9].

#### 3.1.2 Acute Kidney Injury (AKI)

Renal involvement is common, with abnormalities ranging from mild proteinuria to severe AKI in over half of cases in some series. The mechanism of AKI in scrub typhus is mainly due to impaired renal perfusion following volume depletion or increased vascular permeability [10]. Other potential mechanisms include direct tubular toxicity leading to acute tubular necrosis, interstitial nephritis, rhabdomyolysis, and thrombotic microangiopathy secondary to disseminated intravascular coagulation [11]. Renal biopsies have shown mild mesangial hyperplasia, acute tubular necrosis, or tubulointerstitial nephritis [10].

#### 3.1.3 Acute Respiratory Distress Syndrome (ARDS)

ARDS is another severe manifestation, typically arising from systemic vascular inflammation. The major role for the pathogenesis of ARDS associated with scrub typhus is exhibited by capillary leak, direct pulmonary endothelial cell invasion of the organism, and marked iNOS (inducible nitric oxide synthase) expression [12]. Microscopic examination of affected lungs revealed diffuse alveolar damage with hyaline membrane formation and interstitial pneumonitis with infiltration of inflammatory cells, along with *O. tsutsugamushi* antigen deposits in endothelial cells [12]. Factors such as severe infection, anemia, jaundice, and delayed initiation of appropriate antibiotics increase risk [13]. ARDS accounts for a large proportion of organ dysfunction and need for ventilatory support; mortality rates in complicated cases can range from 22% to 45% [14].

#### 3.1.4 Obstructive Jaundice and Hepatic Dysfunction

Liver involvement in scrub typhus is common, presenting as elevated transaminases, hyperbilirubinemia, and sometimes obstructive jaundice [15]. The interplay of vasculitis, sepsis‐induced cholestasis, and bile stasis secondary to acalculous cholecystitis is implicated in the pathogenesis. Jaundice and elevated bilirubin also correlate with higher severity and risk for other organ dysfunctions, including ARDS.

#### 3.1.5 Shock and Multi‐Organ Dysfunction Syndrome (MODS)

Shock in scrub typhus often arises from profound systemic inflammation, vasoplegia, and capillary leak. The vasculopathy and endothelial dysfunction resulting from direct pathogen invasion precipitate circulatory collapse. Multi‐organ dysfunction, involving respiratory, cardiovascular, renal, and hepatic systems, is seen in up to a third of admitted patients with severe disease [16, 17]. Shock requiring vasoactive agents, neurological dysfunction, and renal failure are independent predictors of mortality [17]. The need for multiple supportive interventions and organ support is common, but mortality can be substantially mitigated by early recognition and appropriate antibiotic therapy (e.g., doxycycline), as demonstrated in several cohorts where survival rates were surprisingly good even in the presence of severe MODS [14].

### 3.2 Clinical Implications

- Early Recognition: Scrub typhus must be considered in endemic regions for patients presenting with unexplained febrile illness and evidence of multi‐organ involvement, especially where general abdominal symptoms and atypical presentations like cholecystitis occur.
- Diagnostic Workup: Diagnosis of scrub typhus is mainly confirmed by a positive IgM ELISA and/or pathognomonic eschar with PCR confirmation where feasible [17]. The major diagnostic methods available for laboratory confirmation include identification of the organism in cell culture, antigen detection by immunohistochemical methods, antibody detection by the indirect immunofluorescence assay (IFA), and finding specific nucleic acid targets using molecular methods [16]. Detection of IgM antibody is considered to be diagnostic of an acute infection when compared to IgG antibodies, which suggest a previous infection, especially in endemic areas [16].
- Management: Prompt administration of effective antibiotics (doxycycline/azithromycin/rifampicin/roxithromycin/tetracycline) and critical care support are essential. Doxycycline is the preferred drug in the treatment of scrub typhus [16]. In critically ill patients, including those in shock, the absorption of enterally administered doxycycline may be problematic. In such situations, intravenous doxycycline should be used; where unavailable, intravenous azithromycin may be used [16]. ARDS, AKI, and shock demand aggressive monitoring and multimodal interventions.
- Prognosis: With timely intervention, the prognosis is favorable even in severe cases, but a delay in diagnosis or supportive care substantially increases the mortality risk.

### 3.3 Key Lessons for Clinicians

- In endemic areas, scrub typhus must be a top differential for febrile illness with multi‐organ dysfunction.
- The presence of an eschar is a critical diagnostic clue but may be absent or in an occult location.
- Abdominal symptoms like epigastric pain should prompt consideration of rare complications like acalculous cholecystitis.
- Outcomes are excellent with early administration of appropriate antibiotics (doxycycline) and aggressive supportive care, even in severe cases.

### 3.4 Strengths and Limitations

#### 3.4.1 Strengths

- Rare and complex presentation: Documents a unique and severe multisystem involvement of scrub typhus, including acute kidney injury, ARDS, septic shock, and acalculous cholecystitis, which are scarcely reported.
- Comprehensive clinical assessment: Detailed clinical, laboratory, and imaging data provide a thorough understanding of the disease progression and complications.
- Early diagnosis and effective management: Shows the critical role of timely serological diagnosis and prompt doxycycline‐based treatment combined with intensive supportive care leading to full recovery.
- Educational value: Raises awareness among clinicians in endemic regions to consider atypical and severe presentations for early intervention.

#### 3.4.2 Limitations

- Single case report: Findings cannot be generalized; the report highlights an individual experience without comparing to a larger cohort.
- Limited long‐term follow‐up: Follow‐up is limited to 1 week post‐discharge, so longer‐term sequelae or relapses are unknown.
- Lack of advanced diagnostic tools: The availability of advanced imaging or molecular diagnostic techniques was not mentioned, which might have offered additional insights.
- No detailed microbiological analysis: The strain typing or antibiotic sensitivity of the pathogen was not explored, which could inform treatment strategies.

## 4 Conclusion

This case report highlights a rare and severe presentation of scrub typhus complicated by acute kidney injury, acute respiratory distress syndrome, septic shock, and acalculous cholecystitis. The multiorgan involvement underscores the aggressive systemic vasculitis caused by *Orientia tsutsugamushi* and the potential for rapid clinical deterioration. Early recognition of such atypical and life‐threatening complications is critical, particularly in endemic regions like Nepal, to prompt timely antimicrobial therapy and intensive supportive care. This case emphasizes that clinicians should maintain a high index of suspicion for scrub typhus in patients presenting with multiorgan dysfunction and uncommon abdominal manifestations, as prompt intervention can significantly improve patient outcomes.

## Author Contributions

**Prabhat Kaphle:** conceptualization, formal analysis, investigation, methodology, project administration, software, supervision, validation, visualization, writing – original draft, writing – review and editing. **Maya Upadhyaya:** conceptualization, investigation, methodology, software, supervision, visualization, writing – original draft, writing – review and editing. **Sarjan Shrestha:** conceptualization, investigation, methodology, project administration, resources, supervision, visualization, writing – original draft, writing – review and editing. **Amrita Shrestha:** investigation, methodology, resources, supervision, validation, visualization, writing – original draft, writing – review and editing. **Dhanlaxmi Giri:** investigation, methodology, resources, supervision, validation, writing – original draft, writing – review and editing. **Kamal Hamal:** supervision, writing – original draft, writing – review and editing. **Liladhar Ojha:** supervision, writing – original draft, writing – review and editing.

## Funding

The authors have nothing to report.

## Ethics Statement

Case reports are exempt from ethical approval in our institution.

## Consent

Written informed consent was obtained from the patient for the publication of this case report and any accompanying images. A copy of the written consent is available for review by the editor‐in‐chief of this journal.

## Conflicts of Interest

The authors declare no conflicts of interest.

## Data Availability

The data that support the findings of this study will be available from the corresponding author upon reasonable request.
